# The mechanism of monomer transfer between two structurally distinct PrP oligomers

**DOI:** 10.1371/journal.pone.0180538

**Published:** 2017-07-26

**Authors:** Aurora Armiento, Philippe Moireau, Davy Martin, Nad’a Lepejova, Marie Doumic, Human Rezaei

**Affiliations:** 1 Univ Paris Diderot, Sorbonne Paris Cité, Lab. J.L. Lions UMR CNRS 7598, Inria, Paris, France; 2 Sorbonne Universités, Inria, UPMC Univ Paris 06, Lab. J.L. Lions UMR CNRS 7598, Paris, France; 3 Inria and Université Paris-Saclay, Campus de l’Ecole Polytechnique, 91128 Palaiseau, France; 4 INRA, UR892, Virologie Immunologie Moléculaires, 78350 Jouy-en-Josas, France; 5 Wolfgang Pauli Institute, University of Vienna, Oskar-Morgenstern Platz 1, 1090 Wien, Austria; Scripps Florida, UNITED STATES

## Abstract

In mammals, Prion pathology refers to a class of infectious neuropathologies whose mechanism is based on the self-perpetuation of structural information stored in the pathological conformer. The characterisation of the PrP folding landscape has revealed the existence of a plethora of pathways conducing to the formation of structurally different assemblies with different biological properties. However, the biochemical interconnection between these diverse assemblies remains unclear. The PrP oligomerisation process leads to the formation of neurotoxic and soluble assemblies called O1 oligomers with a high size heterodispersity. By combining the measurements in time of size distribution and average size with kinetic models and data assimilation, we revealed the existence of at least two structurally distinct sets of assemblies, termed *O*^*a*^ and *O*^*b*^, forming O1 assemblies. We propose a kinetic model representing the main processes in prion aggregation pathway: polymerisation, depolymerisation, and disintegration. The two groups interact by exchanging monomers through a disintegration process that increases the size of *O*^*a*^. Our observations suggest that PrP oligomers constitute a highly dynamic population.

## Introduction

**Fig 1:**
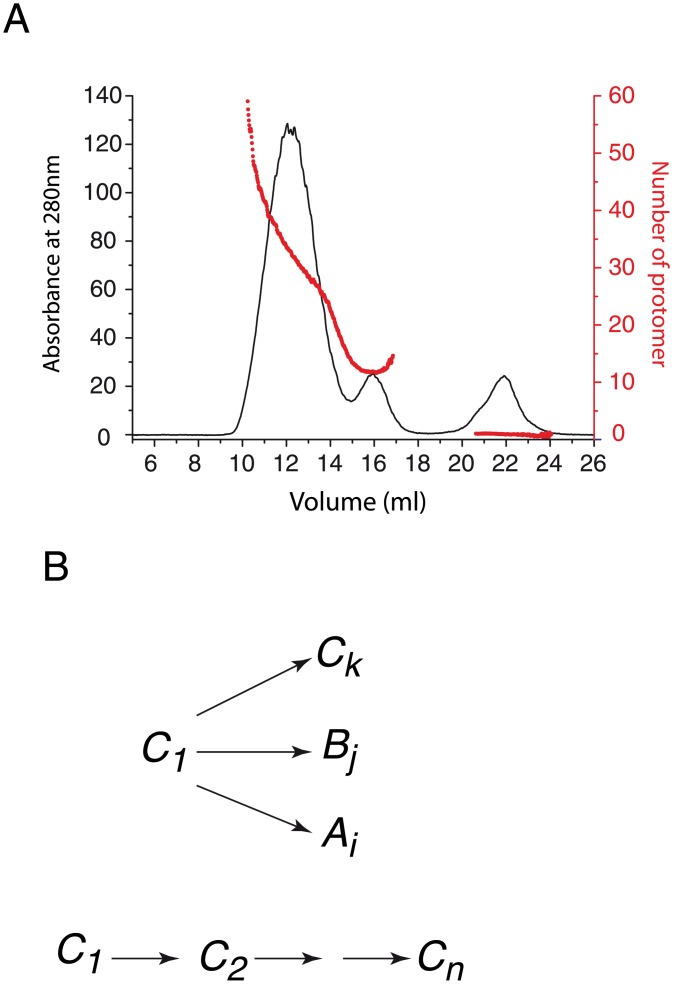
Size distribution of PrP oligomers. **A:** The separation of O1 assemblies from the other type of assemblies and monomer has been performed using size exclusion chromatography coupled to multiwavelength static light scattering lead to estimate size of oligomers generated during PrP oligomerisation. The protein absorbance at 280nm (protein concentration) is represented in black line and size distribution as function of elution volume is in red. **B:** the O1 heterodispersity in size (i.e. molecular weight) could result either from the formation of subpopulations of oligomer (*C*_*k*_, *B*_*j*_ and *A*_*i*_) according to multiple parallel pathways, or from a sequential size increase (from *C*_*n*_ to *C*_*i*_).

Transmissible spongiform encephalopathies (TSEs), or prion diseases, constitute a distinct group of fatal neurodegenerative diseases of humans and other animals. Creutzfeldt-Jakob disease (CJD), Gerstmann-Sträussler-Scheinker syndrome (GSS) and fatal familial insomnia (FFI) are the most common human prion diseases. The prion theory, which has been proposed to describe the self-perpetuation of structural information stored in prion assemblies, is now starting to be extended to a wider range of pathologies caused by protein misfolding and aggregation [1]. One of the intriguing aspects of the prion conversion process is the existence of broad panel of PrP assemblies that are highly heterogeneous in size [2]. The existence of such heterogeneity is associated to stochastic events and often to differences in the micro-environment where the conversion process occurs [3]. However, the diversity in the size of PrP assemblies could also be highly deterministic, as was observed with the oligomerisation process of recombinant PrP (recPrP) in a highly controlled environment [4]. The biochemical and biological implications of such a diversity remain unclear even if structurally different prion assemblies are claimed to be at the basis of the quasi-species phenomenon and prion adaptation to different hosts [5]. The existence of structurally different assemblies raises the question of their respective thermodynamic stability and the consequences of their coexistence in the same environment. Indeed, according to an Ostwald-like ripening phenomenon, the coexistence of assemblies structurally different could lead to a transfer phenomenon from the low stability to the high stability assemblies [6]. The ovine recPrP polymerisation at pH 4.1 and 7.2 leads to the formation of at least three structurally distinct neuro-toxic oligomers [7] whose size and ratio are each governed by the primary structure of PrP [8]. Indeed, at acidic pH the partial unfolding of ovine A136R154Q171 variant of PrP (ARQ) leads to the formation of three distinct oligomers, namely O1, O2 and O3. The biochemical characterisation of these oligomers strongly suggests that their respective folding pathways are different [4]. The O1 oligomers—which constitute the most thermodynamically stable between the three oligomer types—present a heterogeneity in size (Fig 1A).

In this study, we aim at exploring the structural differences between assemblies, if they exist, how they interact and whether this leads to higher stability. We combined depolymerising experiments, followed simultaneously by static light scattering and size exclusion chromatography, with mathematical modelling. Our observation revealed the existence of PrP monomer transfer between coexisting structurally distinct PrP oligomers. This monomer exchange occurs through a disintegration/recapture process which leads finally to select thermodynamically stable assemblies at least in a depolymerising context.

## Materials and methods

### Preparation of recombinant PrP constructs

Full-length Ovine PrP 23-234 (Ala-136, Arg-154, Gln-171 variant) were produced in Escherichia coli and purified as described previously [9]. The O1 oligomers were generated by incubating of OvPrP at 80*mM* at 55°*C* for 6 hours and purified as previously detailed [4] by size exclusion chromatography (SEC). SEC experiments have been performed using TSK 4000 SW column equilibrated with Sodium citrate 20*mM* pH 4.0 coupled to an AKTA Purifier 100 (GE-HealthCare). The size distribution of O1 assemblies was estimated by coupling to multi-wavelength static light scattering with size exclusion chromatography using a TSK 4000SW. The resulting data were transformed to size distribution using a custom MATLAB program. The depolymerisation of O1 assemblies was followed by static light scattering (SLS) by incubating O1 assemblies at 50°*C*.

### Mathematical description of measured quantities

In the following we provide a mathematical description of the quantities measured by SLS and SEC devices. Thanks to these formulas we can compare our qualitative model to the experimental data.

**Fig 2:**
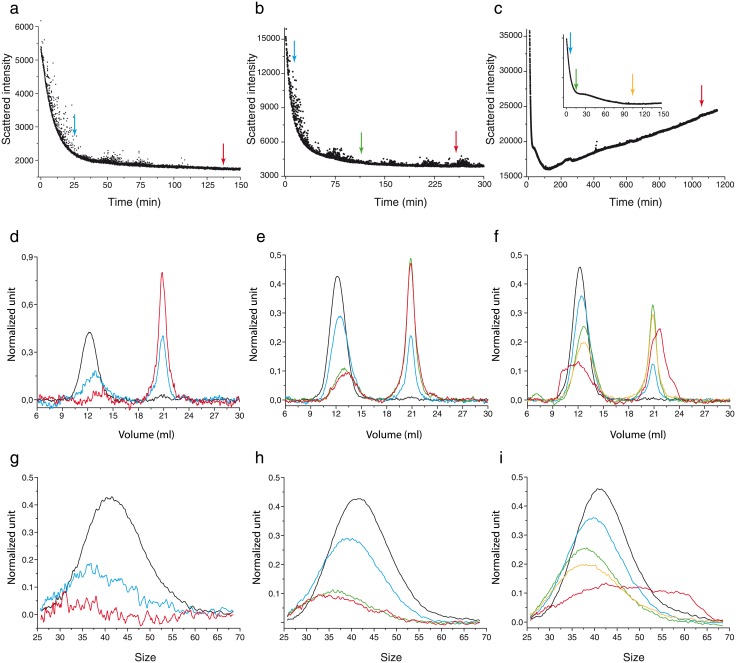
Exploration of O1 oligomers stability through their depolymerisation rate. The depolymerisation rates of O1 assemblies have been explored by using static light scattering (SLS) device, which measures the average molecular weight < *Mw* > as a function of time (**A**, **B** and **C**). Arrows in **A**, **B** and **C** indicate the times of aliquot sampling for SEC (**D**, **E** and **F**) analysis in order to estimate size distribution as a function of time (**G**, **H** and **I**) (see also S1 Appendix). Colours of arrows are associated to the curves colours. Left column (**A**, **D** and **G**) corresponds to depolymerisation experiments performed at O1 concentration of 1*μM*. In **D** and **G** size distribution at times *t* = 0 min (black), *t* = 25 min (blue), *t* = 140 min (red). Middle column (**B**, **E** and **H**) to depolymerisation experiments performed at O1 concentration of 3*μM*. In **E** and **H** size distribution at times *t* = 0 min (black), *t* = 15 min (blue), *t* = 125 min (green), *t* = 270 min (red). Right column (**C**, **F** and **I**) to depolymerisation experiments performed at O1 concentration of 7*μM*. In **E** and **H** size distribution at times *t* = 0 min (black), *t* = 5 min (blue), *t* = 15 min (green), *t* = 95 min (yellow), *t* = 1150 min (red).

The SLS data (Fig 2A, 2B and 2C) measure an affine transform of the mean average molecular weight < *Mw* >, which corresponds mathematically to the second moment of the distribution. Denoting *SLS*(*t*) the measurement by the SLS device at time *t*, we have that there exist two constants such that

$$\begin{array}{c} SLS\left(t\right)=c(m\left(t\right)+\sum_{i=2}^{\infty }i^{2}o_{i}\left(t\right))+c^{\prime }, \end{array}$$ (1)

where *m* is the concentration of isolated monomers, *o*_*i*_ is the concentration of oligomers composed by *i* monomers, and (*c*, *c*′) are two unknown positive constants depending on the experimental setting. We detail in S2 Appendix how we estimate the constants *c* and *c*′, which allows us a quantitative comparison between a simulated kinetics (*m*(*t*), *o*_*i*_(*t*)) and the data.

The SEC data, as we have said before (see S1 Appendix and S1 Fig), can be translated into the size distribution of oligomers (Fig 2G, 2H and 2I). The size distribution data at certain experimental times *t*_*k*_ corresponds to (*o*_*i*_(*t*_*k*_))_*i*>2_.

### Kinetic simulations and data assimilation

Several models have been examined. The ordinary differential equations composing these models have been numerically simulated, using a first-order scheme implemented in MATLAB. We define the oligomer size as the number of monomers composing it and we denote the size unit by “mer” standing for monomer. All assemblies of sizes between 25*mer* and 150*mer* are simulated. We assume that all the oligomers of size 24*mer* disintegrate instantaneously, since none has been experimentally observed. 24*mer*-assemblies thus represent an unstable oligomer structure. The upper bound of 150*mer* has been arbitrarily chosen to encompass all possible oligomer sizes. We remark that SEC data give us the oligomer distribution for sizes between 25 and 70*mer*.

The parameters associated to the experiments into exam were estimated using the Extended Kalman Filter Method [10], implemented in MATLAB, warping the lines of the Verdandi data assimilation library (verdandi.sourceforge.net).

## Results

### Experimental results

We recall that the partial unfolding of ARQ PrP variant leads to the formation of three distinct oligomers: O1, O2 and O3 [4]. We investigate the phenomenon of PrP aggregation by focusing on the study of O1 oligomers. The size distribution analysis of O1 oligomers revealed the existence of highly heterogeneous assemblies regarding their size distribution (Fig 1A). Two hypotheses could explain the heterogeneity in size of O1 oligomers (Fig 1B). The first hypothesis corresponds to the formation of several discrete oligomers through different polymerisation pathways. In this case, each oligomer is structurally different and could have distinct biological properties. The second hypothesis corresponds to a sequential addition of monomers to an oligomer scaffold similar to the nucleation elongation mechanism proposed for amyloid fibril formation. This second hypothesis could generate either structurally equivalent or non-equivalent objects.

In order to discriminate between these two hypotheses and explore the dynamics of the different assemblies forming the O1 peak, we adopted a strategy that consisted of inducing the depolymerisation of O1 assemblies. During the depolymerisation process, the kinetics of size variation were followed in two different ways: by Static Light Scattering (SLS), which reflects the variation of the weight-average molecular weight < *Mw* > (Fig 2A, 2B and 2C), and by size exclusion chromatography (SEC) (Fig 2D, 2E and 2F), which, coupled with multi-wavelength static light scattering (MWLS), gives us access to the size distribution (Fig 2G, 2H and 2I) at different times of the depolymerisation kinetics (See S1 Appendix).

The depolymerisation of O1 assemblies at 1*μM* (equivalent to the total monomer concentration) appears to be total and gives rise to the formation of monomeric PrP as shown by SLS, SEC and size distribution as functions of time (Fig 2A, 2D and 2G). During the depolymerisation process, we observe that the peak decreases, but not in the same way for each size: the concentration of polymers decreases more for large sizes than for smaller sizes (Fig 2G). This asymmetric evolution could suggest either a faster rate of decrease of large O1 assemblies, or could result from the depolymerisation of at least two different species (Fig 1B).

At 3*μM*, we notice a plateau in SLS data (Fig 2B) from 100 min to the end of the experiment and almost no variation between the oligomer distributions at time 125 min and 270 min (Fig 2E and 2H). This suggests that the oligomers system reaches a pseudo-equilibrium.

Moreover, for O1 assemblies at 7*μM*, another particularity was observed. The SLS signal—*i.e.* the average molecular weight < *Mw* > of the system—presents a minimum for *t* = 120 min. During the first step of the process, until time 120 min, < *Mw* > decreases. As the average molecular weight is proportional to the average oligomer size, we can deduce that the system is in a depolymerising/disintegrating mode. For *t* > 120, the SLS signal increases as a function of time, suggesting an increase in the average size of PrP assemblies. This observation is also confirmed by O1 peak profiles in SEC and size distribution data (Fig 2F and 2I) which reveal the formation of high molecular weight assemblies. Two hypotheses could explain the apparition of high molecular weight assemblies at 7*μM*. The first hypothesis corresponds to the formation of *de novo* assemblies formed directly by the monomer. This hypothesis can be immediately discarded as it was previously demonstrated that monomeric ARQ PrP at concentration below 10*μM* is unable to form oligomers in the experiment time-scale [11]. The second hypothesis retained corresponds to an uptake of monomers by thermodynamically stable assemblies. By considering this last hypothesis, a kinetic model based on the depolymerisation/disintegration of O1 assemblies coupled with an uptake of monomer by stable O1 assemblies has been built.

### Aggregation pathway and model design

Equipped with these complementary experimental measurements, we now want to test which chemical reactions are able to explain the system kinetics. To do so, we depart from a pure polymerisation/depolymerisation model. In fact, comparisons between model prediction and experimental data lead us to progressively adapt the model in an iterative manner, as described below. Let us first introduce some useful notations. We denote *M* the free monomers, and *m*(*t*) the concentration of free monomers at time *t*. Similarly, we denote *O*_*i*_ the oligomers composed of *i* monomers, and *o*_*i*_(*t*) the concentration of oligomers of size *i* at time *t*.

The system being closed, any reaction scheme should preserve the total mass denoted by *ρ*, *i.e.* the total concentration of monomers in the system. Specifically, we have

$$\begin{array}{c} \rho =m\left(0\right)+\sum_{i=2}^{\infty }io_{i}\left(0\right)=m\left(t\right)+\sum_{i=2}^{\infty }io_{i}\left(t\right)\;\;\forall \;t>0,. \end{array}$$ (2)

#### Evidence of polymerisation/depolymerisation process

From the experiment at 1*μM* we deduce the existence of a size reducing process. Furthermore, from the pseudo-equilibrium noticed at 3*μM*, we should consider a set of balancing processes. In order to build a kinetic model describing the evolution of the system, we depart from one of the most natural and widespread model. It consists in considering only polymerisation and depolymerisation by monomer addition or loss. The chemical reactions read as follows

$$\begin{array}{rrr} O_{i}+M & \overset{k_{{\text{on}}_{i}}}{\to } & O_{i+1},\\ O_{i} & \overset{k_{{\text{dep}}_{i}}}{\to } & O_{i-1}+M. \end{array}$$

This set of reactions corresponds to the seminal Becker-Döring system [12]

$$\frac{do_{i}}{dt}\;=-m\left(t\right)\left(k_{{\text{on}}_{i}}o_{i}\left(t\right)-k_{{\text{on}}_{i-1}}o_{i-1}\left(t\right)\right)+\left(k_{{\text{dep}}_{i+1}}o_{i+1}\left(t\right)-k_{{\text{dep}}_{i}}o_{i}\left(t\right)\right),\;i\geq 2,$$

$$\frac{dm}{dt}\;=\;\overset{\infty }{\underset{i=2}{\sum }}\left(-m\left(t\right)k_{{\text{on}}_{i}}+k_{{\text{dep}}_{i}}\right)o_{i}\left(t\right).$$

Here, based on previous results [4, 13] as explained above, we do not take into account the spontaneous polymerisation of monomers, taking $k_{{\text{on}}_{1}}=0$. Furthermore, the experiments start with only oligomers, or equivalently *m*(0) = 0, so that polymerisation does not influence the beginning of the reaction.

#### Evidence of a disintegration process

The first thing that we notice is that, considering the SEC data (Fig 2D, 2E and 2F) at the beginning of the reactions, the peak value both slightly shifted to the smaller sizes, lowered, and the polymerised mass decreased. At first sight, this is in line with the dynamics governed by a purely depolymerising system.

At first order, it is known that the Becker-Döring system may be approximated by a transport equation—the so-called Lifshitz-Slyozov system [14, 15]– so that it acts mainly as a drift operator, driving the peak either towards smaller sizes (as observed here at the beginning of the reaction), when depolymerisation is stronger, or towards larger sizes, when polymerisation dominates (as observed at the end of the reaction curve 7*μM*, see Fig 2F). With size-varying coefficients, the model can deform the peak, but polymerised mass can be lost only when polymers reach the smallest stable size. At second order, a correction to the drift operator is given by a diffusion operator [15, 16], leading the peak to be both larger and lower.Therefore, the behaviour of the peaks observed in Fig 2D, 2E and 2F may appear qualitatively plausible at first sight—they both shifted to the left and are more diffuse. However at the beginning of the reactions—where polymerisation is negligible since there is only a very small number of monomers—simulations strongly depart from the data. Indeed, depolymerisation alone could not explain the curve shapes. The correct loss of mass involves a diffusion effect too strong, and a shift towards smaller sizes that is much bigger than the one observed.

**Fig 3:**
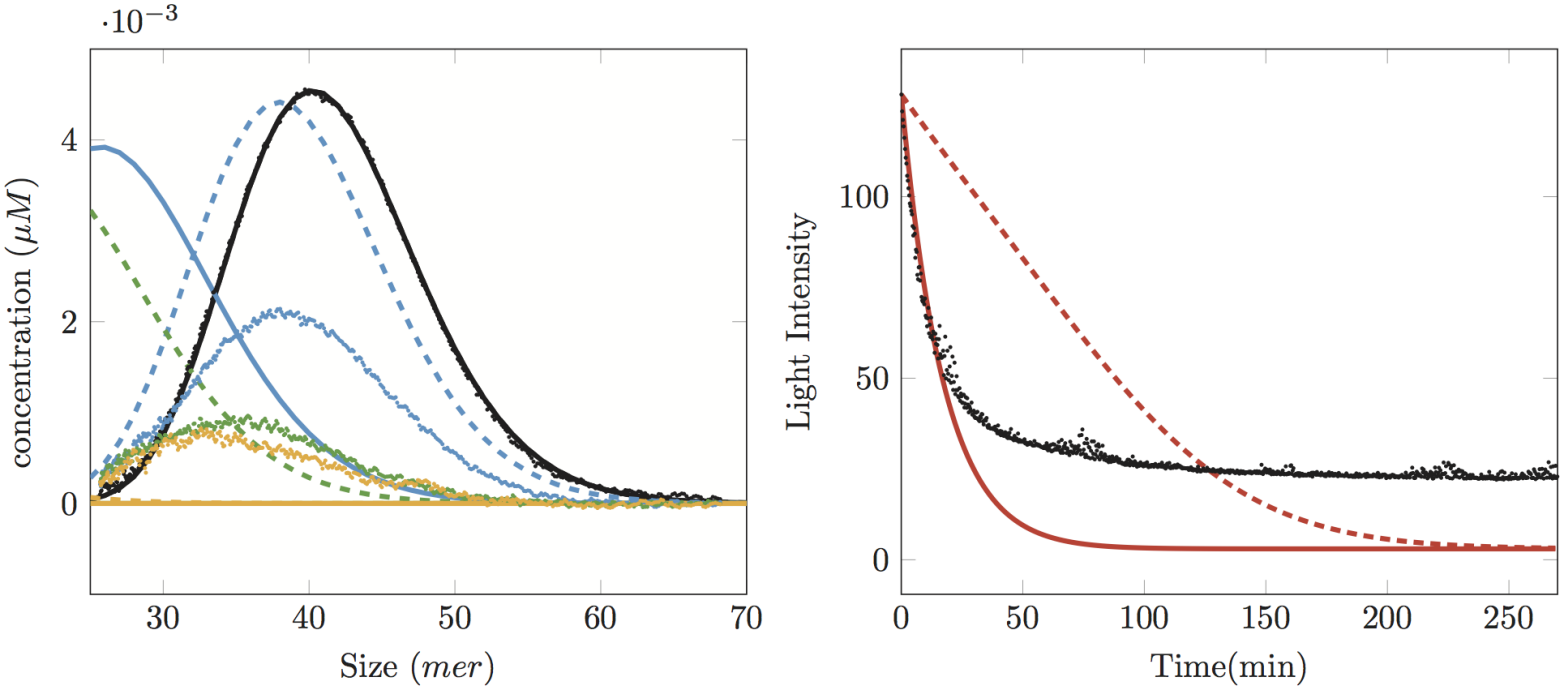
Comparison between experiments (dots) and simulations (dashed and solid lines) with pure depolymerisation models. The initial concentration is 3*μM*. Left: SEC data at times 0 min (black), 15 min (blue), 125 min (green) and 270 min (yellow). Right: SLS data (black dots: experimental, red dashed and solid: simulations). Dashed curves (Left and Right) correspond to *k*_on_ = 0*μM*^−1^. min^−1^, *k*_dep_ = 0.16 min^−1^: the position of the peak for the first time *t* = 15 min is correct for the size distribution, but its height is not and nor is the slope of the SLS data. Solid curves (Left and Right) correspond to *k*_on_ = 0*μM*^−1^. min^−1^, *k*_dep_ = 1 min^−1^: the slope for SLS data fits well at the beginning, but the size-distribution has shifted too much to the left.

For instance, if we take size-independent kinetic rates *k*_on_ = 0*μM*^−1^. min^−1^ and *k*_dep_ = 1 min^−1^, we can see in Fig 3-Right that the solid line fits the SLS data at the beginning of the experiment. However, when we compare the simulated oligomer distribution and the SEC data at *t* = 15 min, we can observe a strong difference both in peak position and in peak value. On the contrary, if we want to approximate the peak position of the distribution at time *t* = 15 min, we consider the parameters *k*_on_ = 0*μM*^−1^. min^−1^ and *k*_dep_ = 0.16 min^−1^, resulting in the dashed lines of Fig 3: the peak position is correct, but its value is much too high, whereas the slope for the SLS data is too small.

This leads us to propose the disintegration process described by the following chemical reactions

$$\begin{array}{c} O_{i}\;\overset{k_{{\text{dis}}_{i}}}{\underset{}{⟶}}\;iM, \end{array}$$

so that we obtain a modified Becker-Döring system

$$\frac{do_{i}}{dt}\;=\;-\left(k_{{\text{on}}_{i}}o_{i}\left(t\right)-k_{{\text{on}}_{i-1}}o_{i-1}\left(t\right)\right)+\left(k_{{\text{dep}}_{i+1}}o_{i+1}\left(t\right)-k_{{\text{dep}}_{i}}o_{i}\left(t\right)\right)-k_{{\text{dis}}_{i}}o_{i},i\geq 2,$$

$$\frac{dm}{dt}\;=\;\overset{\infty }{\underset{i=2}{\sum }}\left(-m\left(t\right)k_{{\text{on}}_{i}}+k_{{\text{dep}}_{i}}+ik_{{\text{dis}}_{i}}\right)o_{i}\left(t\right).$$

#### Evidence of the coexistence of two species

**Fig 4:**
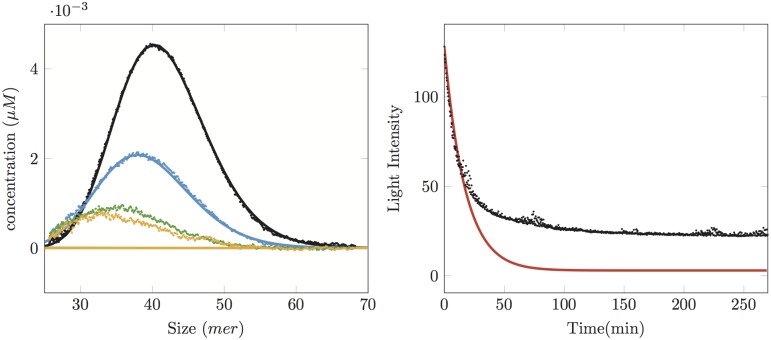
Comparison between experiments (dots) with simulation (solid line) with a polymerisation, depolymerisation and disintegration model. The initial concentration is 3*μM*. Left: SEC data at times 0 (black), 15 min (blue), 125 min (green) and 270 min (yellow). Right: SLS data (black dots: experimental, red solid line: simulations). We see that the simulation data with parameters *k*_on_ = 0*μM*^−1^. min^−1^, *k*_dep_ = 0.16 min^−1^, *k*_dis_ = 0.05 min^−1^ fit well the size distributions and the SLS curve until time 15 min, but afterwards, due to the disintegration process, they all go to zero, in contrast to the experimental measurements.

With this additional disintegration term, the beginning of the reaction is in good agreement with the data as shown in Fig 4. However, the disintegration term leads any size of polymer to vanish exponentially fast at a rate *k*_dis_, even if there is polymerisation.

This behaviour is illustrated in Fig 4: choosing the size-independent kinetic parameters *k*_on_ = 0*μM*^−1^. min^−1^, *k*_dep_ = 0.16 min^−1^, *k*_dis_ = 0.05 min^−1^, we are able to fit the beginning of the 3*μM* experiment both in SLS data and in SEC data. The model cannot reproduce the long-time behaviour (*t* > 20 min) because the simulated oligomer distribution tends to zero too rapidly.

Therefore with this model, we can well describe the experiments at the initial concentration of 1*μM*, since almost all oligomers disappear, but neither the long-time behaviour at 3*μM* nor the recapture process observed at the end of the reaction at 7*μM* may be simulated.

To overcome this paradox, we propose the existence of at least two structurally distinct species coexisting under the O1 peak: one unstable, subject to disintegration and with very small polymerisation and depolymerisation rates (we denote this species *O*^*b*^, and $O_{i}^{b}$ the oligomers of this type containing *i* monomers), the other more stable, with a disintegration rate very low (we denote this species *O*^*a*^, and $O_{i}^{a}$ the oligomers of this type containing *i* monomers). Gathering all these elements, the simplest possible model consists in the following three types of reactions

$$\begin{array}{ccc} O_{i}^{a}+M & \overset{k_{{\text{on}}_{i}}}{\to } & O_{i+1}^{a},\\ O_{i}^{a} & \overset{k_{{\text{dep}}_{i}}}{\to } & O_{i-1}^{a}+M,\\ O_{i}^{b} & \overset{k_{{\text{dis}}_{i}}}{\to } & iM, \end{array}$$

which result in the following differential system

$$\frac{do_{i}^{a}}{dt}\;=\;m\left(k_{{\text{on}}_{i-1}}o_{i-1}^{a}-k_{{\text{on}}_{i}}o_{i}^{a}\right)-\left(k_{{\text{dep}}_{i}}o_{i}^{a}-k_{{\text{dep}}_{i+1}}o_{i+1}^{a}\right),\;i\geq 2,$$ (3)

$$\frac{do_{i}^{b}}{dt}\;=\;-k_{{\text{dis}}_{i}}o_{i}^{b},\;\;i\geq 2,$$ (4)

$$\frac{dm}{dt}\;=\;-m\overset{\infty }{\underset{i=2}{\sum }}k_{{\text{on}}_{i}}o_{i}^{a}+\overset{\infty }{\underset{i=2}{\sum }}k_{{\text{dep}}_{i}}o_{i}^{a}+\overset{\infty }{\underset{i=2}{\sum }}ik_{{\text{dis}}_{i}}o_{i}^{b},$$ (5)

$$m\left(0\right)\;=\;0,\overset{\infty }{\underset{i=2}{\sum }}io_{i}^{a}\left(0\right)+io_{i}^{b}\left(0\right)=\rho ,$$ (6)

where $o_{i}^{a}\left(t\right)$ and $o_{i}^{b}\left(t\right)$ denote the concentrations at time *t* of the stable oligomers of size *i* and the unstable oligomers of size *i*, respectively, with $o_{i}\left(t\right)=o_{i}^{a}\left(t\right)+o_{i}^{b}\left(t\right).$ Summing the equations of the system yields the following mass balance

$$\begin{array}{c} m\left(t\right)+\sum_{i=2}^{\infty }i\left(o_{i}^{a}\left(t\right)+o_{i}^{b}\left(t\right)\right)=\rho ,\;\forall \;t>0. \end{array}$$

We do not make the system any more complex and show in the next section that it is, in fact, sufficient to quantitatively explain the experimental results.

### Model/Data quantitative adjustment

Until now, the entire analysis was carried out qualitatively, by iterating direct simulations. At this point, having an already qualitatively good agreement between the simulations outputs and the experimental records, we can envision to register quantitatively the model with the data by identifying the best parameters explaining the experimental measurements.

To avoid any overfitting, we choose constant disintegration, polymerisation and depolymerisation rates, denoted by *k*_dis_, *k*_on_ and *k*_dep_ respectively. We recall that we do not consider polymerisation and depolymerisation for the unstable species. We are thus led to estimate only four parameters: the three reaction rates, and the ratio of stable oligomers $\frac{o_{i}^{a}}{o_{i}^{a}+o_{i}^{b}}$ at the initial time, which we also assume to be independent of the size *i*.

### Parameter identification based on Kalman estimation

To estimate the four parameters *k*_on_, *k*_dep_, *k*_dis_ and $\theta =\frac{o_{i}^{a}\left(0\right)}{o_{i}^{a}\left(0\right)+o_{i}^{b}\left(0\right)}$, we rely on an Extended Kalman Filter (EKF) approach [10] implemented in MATLAB. In order to apply the EKF, we need to define a complete dynamical system including the parameters to be estimated. To this end, the variables $o_{i}^{b}$ are substituted by their analytical expressions $o_{i}^{b}\left(t\right)=e^{-k_{\text{dis}}t}o_{i}^{b}\left(0\right)=e^{-k_{\text{dis}}t}{o_{i}^{b}}_{0}$. We consider the state variables $o_{i}^{a}$, ${o_{i}^{b}}_{0}$ and *m*, extended with the parameters *k*_on_, *k*_dep_ and *k*_dis_ with a null dynamics

$$\frac{d}{dt}k_{\text{on}}\;=\;0,\;\frac{d}{dt}k_{\text{dep}}\;=0,\;\frac{d}{dt}k_{\text{dis}}\;=\;0.$$

We also need to define an *a priori* of the initial state that would be the initial condition of the EKF estimator. Direct model simulations allowed us to define the *a priori* values $k_{{\text{on}}_{0}}=0.031\min^{-1}\mu M^{-1}$, $k_{{\text{dep}}_{0}}=0.09\min^{-1}$, $k_{{\text{dis}}_{0}}=0.07\min^{-1}$, *θ*_0_ = 0.2 of the parameters *k*_on_, *k*_dep_, *k*_dis_ and *θ* respectively. Having measured experimentally *o*_*i*_(0) with the SEC data (see S1 Appendix and S2 Fig for more details), the EKF initial condition for ($o_{i}^{a}$, $o_{i}^{b}{}_{0}$, *m*, *k*_on_, *k*_dep_, *k*_dis_) is thus fixed to

$$\begin{array}{c} (\theta_{0}o_{i}\left(0\right),\left(1-\theta_{0}\right)o_{i}\left(0\right),0,k_{{\text{on}}_{0}},k_{{\text{dep}}_{0}},k_{{\text{dis}}_{0}}). \end{array}$$

The EKF estimator is a sequential estimator estimating the trajectory in time. Its dynamics results in the contribution of two terms:

- the model—that summarises our knowledge on the oligomer system,
- a corrective term exploiting the availability of some observation on the system.

The weight of each term is balanced by a gain operator—the Kalman gain—based on an estimation error covariance in the model and in the data. We call *W* the estimation of the observation noise covariance and $P_{k_{\text{on}}}$, $P_{k_{\text{dep}}}$, $P_{k_{\text{dis}}}$, *P_θ_* the initial uncertainty covariance of the model parameters. Here, we decide to only rely on the SLS data—the observation operator being the second moment as in [17]—during the estimation. The SEC data are then used to validate the resulting estimations.

As our model is non-linear, we cannot imagine to prescribe a too-large initial uncertainty covariance for the initial parameters. By consequence, we choose to iterate the EKF estimation by restarting the estimator from the previous estimated result while keeping the same initial covariance. This approach is known as the Iterative Extented Kalman Filter (IEKF) approach [18]. We stop the iterations when, for all parameters, the absolute value of the difference between two successive estimations is less than a threshold set to 10^−5^, hence in our case, a relative error of less than 1%.

**Fig 5:**
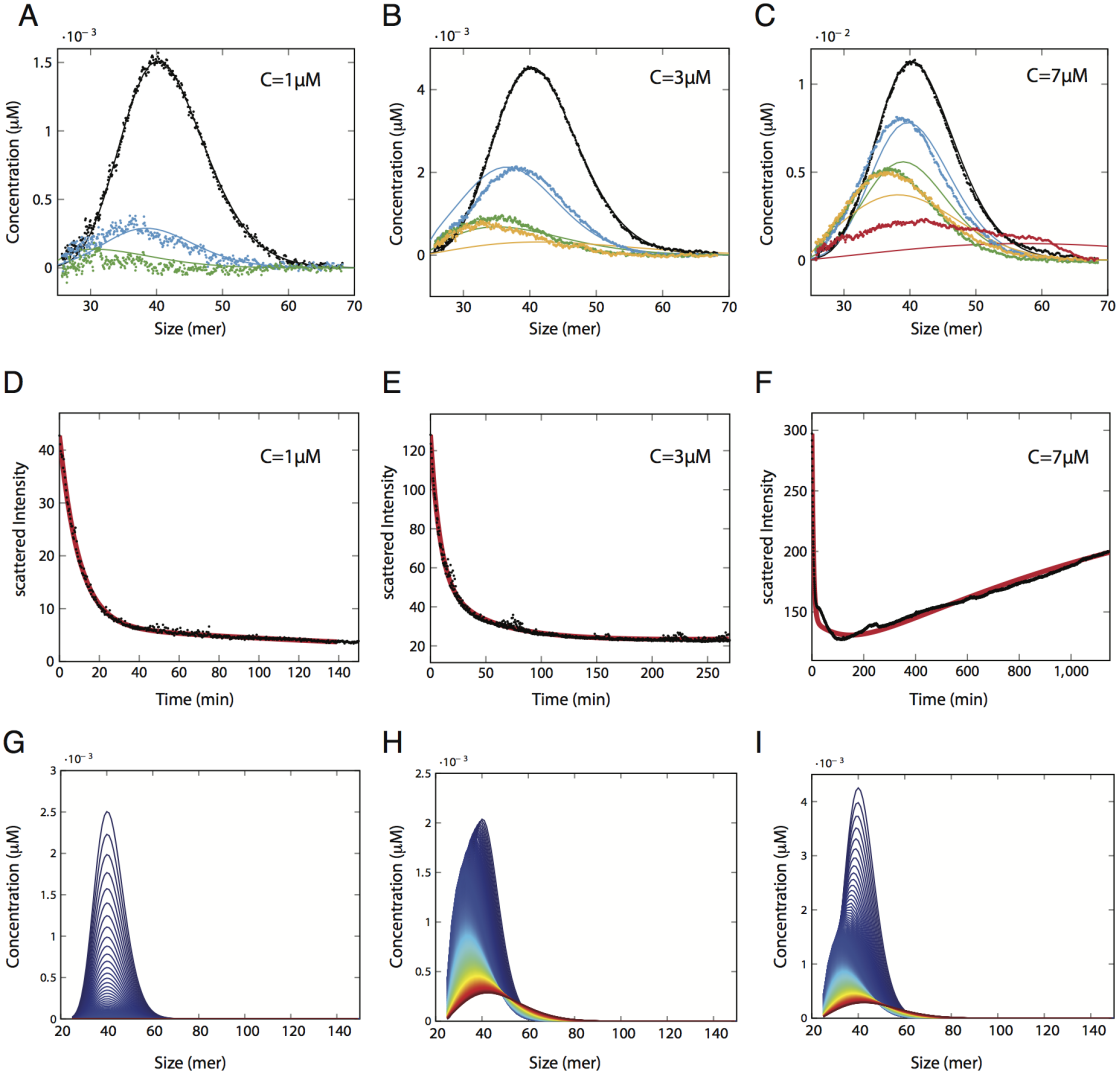
Comparison between experimental data and synthetic observations. Size distribution (**A**, **B** and **C**) and light scattering intensity (**D**, **E** and **F**) as a function of time, at O1 concentration *ρ* = 1, 3 and 7*μM*, have been fitted (blacksolid lines in **A**, **B** and **C**, solid red line in **D**, **E** and **F**) using best-fit parameters reported in Table 1. The experimental data are represented in dots black of the same colour as the simulation in **A**, **B** and **C**, in black in (**D**, **E** and **F**). Simulation corresponding to the evolution of size distribution of $o_{i}^{b}$ (**G**) and $o_{i}^{a}$ (**H**) and the sum $o_{i}^{a}+o_{i}^{b}$ (**I**).

Best-fit parameters are reported in Table 1 and the comparison between model simulation and experimental data is shown in Fig 5. For such a simple model, we found a remarkable quantitative agreement, as well as parameters remaining in the same order of magnitude. Moreover, the remarkable agreement obtained between the experimental size distribution and those predicted by the estimated parameters on SLS leads us to validate the monomer exchange model between *O*^*a*^ and *O*^*b*^, while other models failed to fit the time-evolution of oligomer size distribution.

**Table 1:** Best-fit parameters obtained by the data assimilation method on the two-species model *O*^*a*^ and *O*^*b*^ (for details see also SI). *ρ* corresponds to the total monomer concentration.

	*k*_on_ (*μM*^−1^. min^−1^)	*k*_dep_ (min^−1^)	*k*_dis_ (min^−1^)	$\frac{o_{i}^{a}}{o_{i}^{a}+o_{i}^{b}}$
*ρ* = 1*μM*	0.169	0.228	0.102	0.147
*ρ* = 3*μM*	0.236	0.602	0.127	0.478
*ρ* = 7*μM*	0.060	0.242	0.152	0.491

These observations lead us to validate the monomer exchange model between the two sets of O1 assemblies. Our conclusion is thus twofold. First, a very simple two-species model is able to fit the data, whereas a one-species model, even with size-dependent coefficients, is not. Second, we surprisingly do not need size-dependent coefficients (see Table 1) to obtain an acceptable data-measurement agreement (see Fig 5). This means that it is possible that within a given species the objects of different sizes are structurally equivalent.

### Robustness of parameter estimation

**Fig 6:**
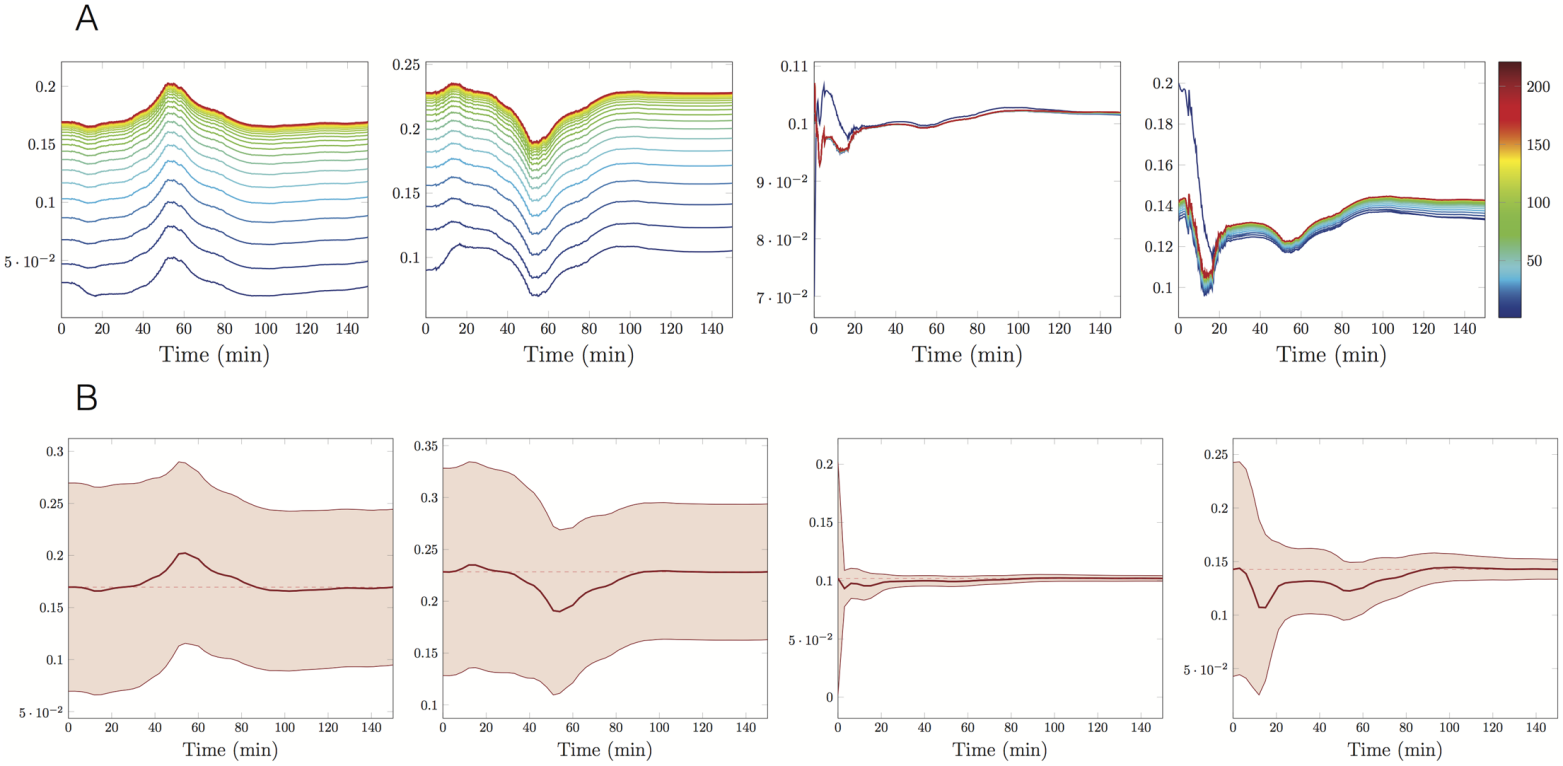
Time evolution of the uncertainty on the estimations, ρ=1μM. *A priori* $k_{{\text{on}}_{0}}=0.031\min^{-1}\mu M^{-1}$, $k_{{\text{dep}}_{0}}=0.09\min^{-1}$, $k_{{\text{dis}}_{0}}=0.07\min^{-1}$, *θ*_0_ = 0.2, observation covariance *W* = 10, predicted standard deviation of initial *a priori* estimations as in Table 2. **A:** One curve every ten iterations of the Extended Kalman method, number of the iteration in colorscale. **B:** Trajectories of the final estimators (red solid lines), the final estimations (red dashed lines) and 95% prediction uncertainty intervals (red areas). From the left to the right estimators of *k*_on_, *k*_dep_, *k*_dis_ and *θ*.

**Fig 7:**
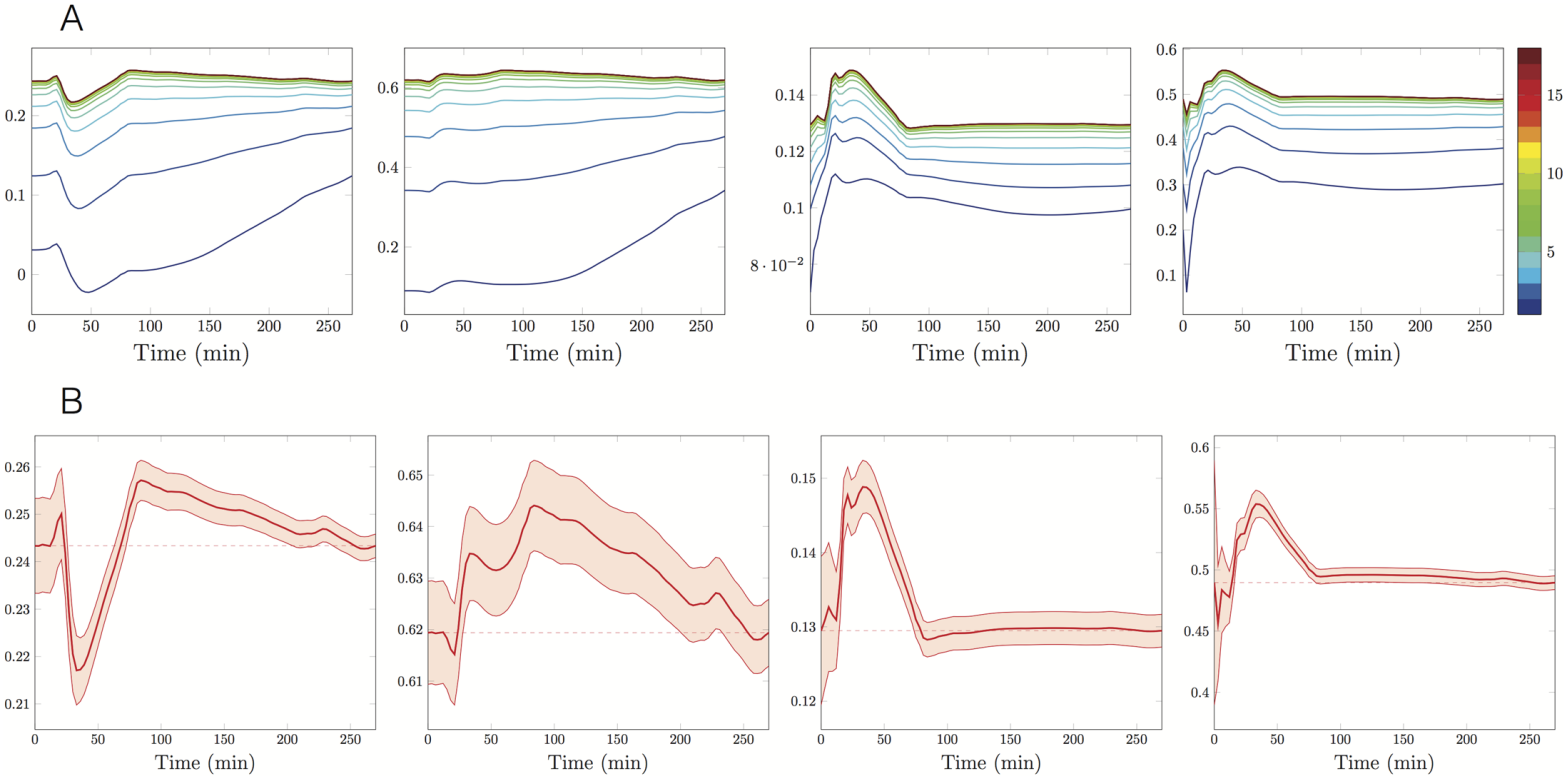
Time evolution of the uncertainty on the estimations, ρ=3μM. *A priori* $k_{{\text{on}}_{0}}=0.031\min^{-1}\mu M^{-1}$, $k_{{\text{dep}}_{0}}=0.09\min^{-1}$, $k_{{\text{dis}}_{0}}=0.07\min^{-1}$, *θ*_0_ = 0.2, observation covariance *W* = 8, predicted standard deviation of initial *a priori* estimations as in Table 2. **A:** 19 iterations of the Extended Kalman method, number of the iteration in colorscale. **B:** Trajectories of the final estimators (red lines), the final estimations (red dashed lines) and 95% prediction uncertainty intervals (red areas). From the left to the right estimators of *k*_on_, *k*_dep_, *k*_dis_ and *θ*.

**Fig 8:**
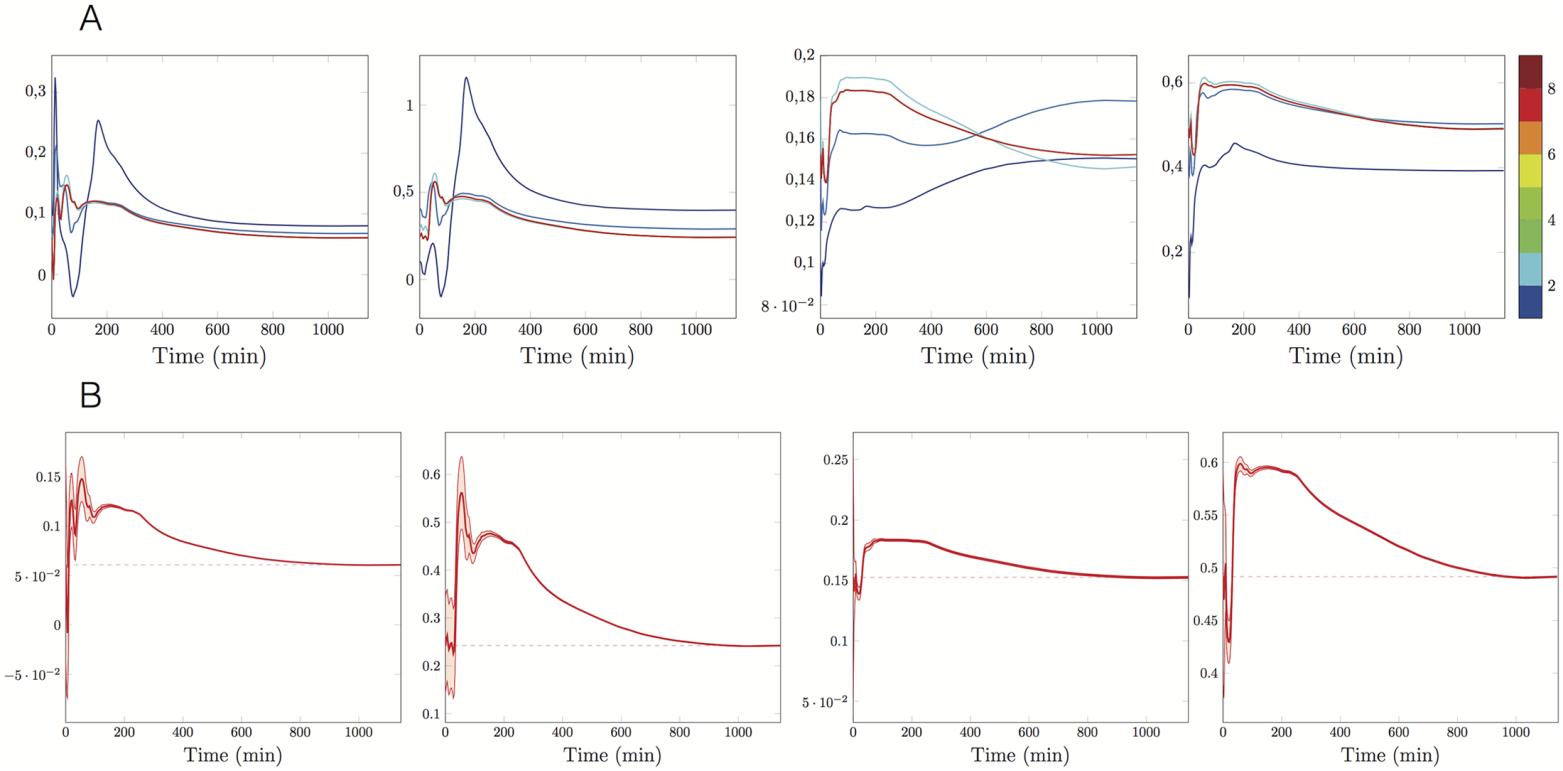
Time evolution of the uncertainty on the estimations, ρ=7μM. *A priori* $k_{{\text{on}}_{0}}=0.1\min^{-1}\mu M^{-1}$, $k_{{\text{dep}}_{0}}=0.1\min^{-1}$, $k_{{\text{dis}}_{0}}=0.1\min^{-1}$, *θ*_0_ = 0.1, observation covariance *W* = 10, predicted standard deviation of initial *a priori* estimations as in Table 2. **A:** 9 iterations of the Extended Kalman method black, number of the iteration in colorscale. **B:** Trajectories of the final estimators (red lines), the final estimations (red dashed lines) and 95% prediction uncertainty intervals (red areas). From the left to the right estimators of *k*_on_, *k*_dep_, *k*_dis_ and *θ*.

When using the EKF, we also estimate the standard deviation of the estimation error over time. Therefore, in Figs 6A, 7A and 8A we present the time evolution of the estimated parameters mean values relative to the experiments at 1,3 and 7*μM*, respectively, whereas in Figs 6B, 7B and 8B, we show both the evolution of the parameter estimators ${\hat{k}}_{on}$, ${\hat{k}}_{dep}$, ${\hat{k}}_{dis}$, $\hat{\theta }$ at the last iteration of the IEK method and the associated 95% predicted uncertainty intervals. In all the cases, as expected, the uncertainty decreases in time. The initial and final values of the error standard deviations are reported in Table 2.

**Table 2:** Standard deviations (StD) of the parameter estimation errors.

*ρ* = 1*μM*	*k*_on_	*k*_dep_	*k*_dis_	*θ*
Initial StD	10^−1^	10^−1^	10^−1^	10^−1^
Final StD	7.1 * 10^−2^	6.5 * 10^−2^	2.4 * 10^−3^	9.3 * 10^−3^
*ρ* = 3*μM*	*k*_on_	*k*_dep_	*k*_dis_	*θ*
Initial StD	10^−2^	10^−2^	10^−2^	10^−1^
Final StD	2.5 * 10^−3^	6.5 * 10^−3^	2.1 * 10^−3^	5.6 * 10^−3^
*ρ* = 7*μM*	*k*_on_	*k*_dep_	*k*_dis_	*θ*
Initial StD	10^−1^	10^−1^	10^−1^	10^−1^
Final StD	2 * 10^−4^	9.5 * 10^−4^	8.5 * 10^−4^	7.4 * 10^−4^

More precisely, in Fig 6B, we notice that the uncertainty region around the estimator of the disintegration parameter quickly reduces and then keeps a stable spread. We deduce that the parameter *k*_dis_ is well-identifiable. On the contrary, the parameters *k*_on_ and *k*_dep_ are not well-identifiable. Analogously, at *ρ* = 7*μM* the rapid narrowing of all the four uncertainty regions (Fig 8B) suggests that all the parameters are well identifiable.

Another comment should be emphasised about the estimated parameter trajectory reported in Figs 6A, 7A and 8A. As the EKF is sequential, the estimated parameters have a trajectory aiming at targeting the true kinetic parameters. When considering the successive iterations of the IEKF, we see as expected that the variations of the estimators are reduced. However smaller variations remain after reaching the convergence threshold. These variations typically indicate a remaining model error of small amplitude and provide an insight on the order of magnitude of the terms neglected in the model.

## Discussion

Our step-by-step approach, from experimental analysis to data assimilation, leads us to a partly counter-intuitive conclusion: the existence of monomer exchange between two types of PrP oligomer assemblies. The formation of heterodisperse assemblies during the evolution of pathologies due to protein misassembly raises the question of their coexistence and their evolution. This phenomenon occurs during prion conversion for which several species could coexist and form what is also commonly called prion quasi-species [19, 20]. From a thermodynamical point of view, it is clear that not all assemblies are kinetically and energetically equivalent and some species with specific biological activities could be generated transitorily. However, the evolution of all these assemblies should follow specific thermodynamic and kinetic rules such as selection by higher stability and/or higher rate of formation. In the present work we demonstrate that there exist at least two types of oligomers which are simultaneously generated from monomeric PrP. In conditions that could biologically correspond to monomer depletion, we demonstrate that these two oligomer species are able to exchange monomers. The biological consequences of such a phenomenon could be the transitory apparition of physiopathological patterns and the existence of buffer assemblies serving as monomer reservoirs to enhance and maintain more stable assemblies. It is also clear that such a phenomenon should be considered for all therapeutic purposes.

**Fig 9:**
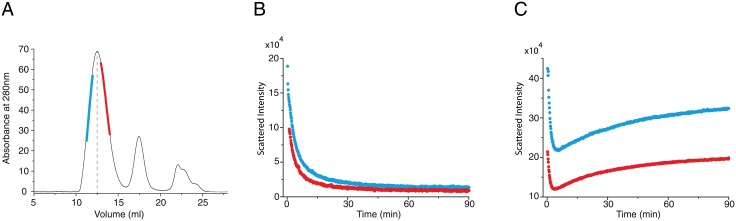
Experimental investigation of the size invariance of $\frac{o_{i}^{a}}{o_{i}^{a}+o_{i}^{b}}$. **A:** asymmetric purification of O1 assemblies. The blue and red lines indicate purified portions of O1 peak corresponding to large and small assemblies, respectively. The depolymerisation of large O1 assemblies (in blue) and small O1 assemblies (in red) has been monitored at 0.3*μM* **(B)** and 6*μM* **(C)** and reveals an isokinetic behaviour between large and small O1 assemblies. This isokinetic behaviour suggests a kinetic parameter invariance all along the size distribution landscape and sheds light on the molecular basis of O1 architecture.

The elaboration of kinetic pathways and simulations describing the exchange process assumed that the kinetic constants (*k*_dis_, *k*_dep_ and *k*_on_) do not depend on the size of PrP assemblies. black Another important assumption is the size invariance of the ratio of *O*^*a*^ over *O*^*a*^ + *O*^*b*^ at *t*_0_. However, it is expected that *O*^*a*^– as stable assemblies—populates higher molecular weight assemblies when unstable assemblies as *O*^*b*^—less represented in the distribution—remains as small objects. The experimental investigation of the size invariance of *o*^*a*^ over *o*^*a*^ + *o*^*b*^ and kinetic constants (*k*_dis_, *k*_dep_ and *k*_on_) confirms the validity of this assumption—as illustrated in Fig 9—and challenges the conventional structural model of PrP oligomer assemblies arguing in favour of an entanglement of kinetic pathway of the formation of *O*^*a*^ and *O*^*b*^ assemblies.

## Supporting information

S1 Appendix Details on the size exclusion chromatography (SEC) scales. (PDF)

S1 Fig Multi-wavelength static light scattering data, allowing us to have a correspondence between the elution volume measured by SEC (in *ml*) and the size of the polymers going through the SEC device (in the number of monomers, denoted *mer*). (EPS)

S2 Fig Normalised initial distribution for the three experiments considered, at 1*μM* (dark blue), 3*μM* (red) and 7*μM* (green). We can observe that they superimpose very well.
(EPS)

S2 Appendix Details on the static light scattering (SLS) scales. (PDF)

S3 Fig Noise measurement of the empty cuvette for the SLS device. (EPS)

S4 Fig Comparison between SLS (black dots) data and second moment computed from SEC data (red points) for the three experiments, from left to right: 1*μM*, 3*μM* and 7*μM*. (EPS)
