# Supplementary material for: The mechanism of monomer transfer between two structurally distinct PrP oligomers
Source: PLoS One. 2017 Jul 26;12(7):e0180538. doi: 10.1371/journal.pone.0180538 (PMC5528842; doi:10.1371/journal.pone.0180538)
Supplement: S1 Appendix — (PDF) [file pone.0180538.s001.pdf]

The mechanism of monomer transfer between two structurally distinct PrP oligomers

Aurora Armiento [1], Philippe Moireau [3◕*], Davy Martin [4], Nad'a Lepejova [4], Marie Doumic [2,5◕*], Human Rezaei[4◕*],

1 Univ Paris Diderot, Sorbonne Paris Cité, Lab. J.L. Lions UMR CNRS 7598, Inria, Paris, France
2 Sorbonne Universités, Inria, UPMC Univ Paris 06, Lab. J.L. Lions UMR CNRS 7598, Paris, France
3 Inria and Université Paris-Saclay, Campus de l'Ecole Polytechnique, 91128 Palaiseau, France
4 INRA, UR892, Virologie Immunologie Moléculaires, 78350 Jouy-en-Josas, France
5 Wolfgang Pauli Institute, University of Vienna, Oskar-Morgenstern Platz 1, 1090 Wien, Austria

◕These authors contributed equally to supervise this work.
* philippe.moireau@inria.fr, marie.doumic@inria.fr, human.rezaei@jouy.inra.fr

S1 Appendix.

Details on the Size Exclusion Chromatography (SEC) scales. The aggregates pass through a gel grid, and the device measures the concentration of molecules associated to the same elution volume. In Fig. S1, we see the correspondence between the volume measurement and the size of a polymer. We use this scale to obtain a measurement of the size distribution, up to a constant to be determined. In Fig. S2, we scaled the initial oligomer size distribution of each experiment to have their peak value equal to one: they exhibited a remarkable agreement, which led us to have a high level of confidence in initial size distribution data. For each experiment, we then scaled this initial size distribution measurement by the known initial concentration (1, 3 and $7\mu M$ respectively), and used the same factor to scale the measurements at the following times.
