# Supplementary material for: The mechanism of monomer transfer between two structurally distinct PrP oligomers
Source: PLoS One. 2017 Jul 26;12(7):e0180538. doi: 10.1371/journal.pone.0180538 (PMC5528842; doi:10.1371/journal.pone.0180538)
Supplement: S2 Appendix — (PDF) [file pone.0180538.s004.pdf]

The mechanism of monomer transfer between two structurally distinct PrP oligomers

Aurora Armiento [1], Philippe Moireau [3☯*], Davy Martin [4], Nad'a Lepejova [4], Marie Doumic [2,5☯*], Human Rezaei[4☯*],

1 Univ Paris Diderot, Sorbonne Paris Cité, Lab. J.L. Lions UMR CNRS 7598, Inria, Paris, France
2 Sorbonne Universités, Inria, UPMC Univ Paris 06, Lab. J.L. Lions UMR CNRS 7598, Paris, France
3 Inria and Université Paris-Saclay, Campus de l'Ecole Polytechnique, 91128 Palaiseau, France
4 INRA, UR892, Virologie Immunologie Moléculaires, 78350 Jouy-en-Josas, France
5 Wolfgang Pauli Institute, University of Vienna, Oskar-Morgenstern Platz 1, 1090 Wien, Austria

☯These authors contributed equally to supervise this work.
* philippe.moireau@inria.fr, marie.doumic@inria.fr, human.rezaei@jouy.inra.fr

S2 Appendix.

Details on the Static Light Scattering (SLS) scales. As said in the Main Text, the Static Light Scattering (SLS) measures an affine transformation of what is mathematically called the second moment of the polymer concentration, i.e. the quantity $m(t) + \sum_{i\geq 2} i^2 o_i(t)$. Denoting $SLS(t)$ the experimental measurement of the SLS at time t, we have, for two constants $c > 0$ and $c' > 0$,

$$SLS(t) = c\left(m(t) + \sum_{i=2}^{\infty} i^2 o_i(t)\right) + c'. \tag{1}$$

To compare the simulations to the data, we thus have to estimate c and c'. We proceed as follows:

- The constant c' corresponds to the mean amplitude of the noise measured in a cuvette containing no protein (See Fig S3). We measured $c' = 1393$ (in Light Intensity).
- To estimate the constant c, several methods are possible. After testing several, and evaluating the confidence we may have in each, the best appeared to use the initial SEC measurements to estimate $m(0) + \sum_{i=2}^{\infty} i^2 o_i(0)$, and then take this value to calculate c such that

$$SLS(0) = c\left(m(0) + \sum_{i=2}^{\infty} i^2 o_i(0)\right) + c'.$$

This gave us one value of c for each experiment, namely 93 ($1\mu M$), 109 ($3\mu M$) and 114 ($7\mu M$). We chose the mean of these values, $c = 105$, which moreover gave a good time-dependent agreement between SLS and SEC data (see Fig S4).
